# Myofascial Release of the Hamstrings Improves Physical Performance—A Study of Young Adults

**DOI:** 10.3390/healthcare9060674

**Published:** 2021-06-04

**Authors:** Keisuke Itotani, Kanta Kawahata, Wakana Takashima, Wakana Mita, Hitomi Minematsu, Hiroyuki Fujita

**Affiliations:** 1 Department of General Rehabilitation, Faculty of Allied Health Sciences, Yamato University, 2-5-1 Katayama-cho, Suita City, Osaka 564-0082, Japan; kk17221010@yamato-univ.jp (K.K.); wt17221026@yamato-univ.jp (W.T.); wm17221043@yamato-univ.jp (W.M.); hm17221044@yamato-univ.jp (H.M.); 2 Department of Physical Therapy, Faculty of Health Care, Osaka University of Human Sciences, 1-4-1 Shoujaku, Settsu City, Osaka 566-8510, Japan; h-fujita@kun.ohs.ac.jp

**Keywords:** myofascial release, physical performance, maximum walking speed, standing long jump

## Abstract

Physical performance is mainly assessed in terms of gait speed, chair rise capacity, and balance skills, and assessments are often carried out on the lower limbs. Such physical performance is largely influenced by the strength of the quadriceps and hamstrings muscles. Flexibility of the hamstrings is important because quadriceps muscle activity influences the hip flexion angle. Therefore, hamstring flexibility is essential to improve physical performance. In this study, Myofascial Release (MFR) was applied to the hamstrings to evaluate its effects. MFR on the hamstrings was performed on 17 young adults. Physical function and physical performance were measured before, immediately after, and 5 days after the MFR intervention: finger floor distance (FFD), range of motion (ROM) of the straight leg raising test (SLR), standing long jump (SLJ), squat jump (SJ), functional reach test (FRT), comfortable walking speeds (C-walking speed), and maximum walking speeds (M-walking speed). The results of the analysis show a significant increase in FFD (−2.6 ± 8.9 vs. 0.4 ± 9.4 vs. 2.4 ± 8.9, *p* < 0.01), SLJ (185.6 ± 44.5 vs. 185.0 ± 41.8 vs. 196.6 ± 40.1, *p* < 0.01), and M-walking speed (2.9 ± 0.6 vs. 3.0 ± 0.6 vs. 3.3 ± 0.6, *p* < 0.01). This study has shown that MFR for hamstrings not only improves flexibility but also increases M-walking speed and physical performance of the SLJ. As MFR is safe and does not involve joint movement, it may be useful for maintaining and improving performance and flexibility during inactivity and for stretching before exercise.

## 1. Introduction

In 2020, COVID-19 had an impact on the activities of people around the world. Maugeri et al. [1] state that the pandemic caused by COVID-19 restricted the activities of many people and that isolation itself led to a decrease in physical activity. Physical inactivity has adverse health effects such as obesity, muscle atrophy, and bone loss [2]. In addition, a decrease in muscle strength due to inactivity may initiate a vicious cycle of inactivity, eventually resulting in impaired health. According to Shang et al. [3], in pandemic-affected areas, hospital beds are limited and overwhelmed by the large numbers of patients, making it difficult for those without COVID-19 to receive regular medical care. Therefore, the importance of preventive medicine needs to be reaffirmed. Shang et al. [3] reported that the rapidly increasing number of COVID-19 cases was posing a huge challenge to medical systems worldwide. In pandemic-affected areas, hospital beds may be limited and overwhelmed by the large numbers of patients. The pandemic may consistently contribute to the shortage of medical resources and ultimately collapse medical systems. In such circumstances, it is valuable to provide methods to prevent inactivity that do not require too much direct medical care and that can be carried out at home. Falvey et al. [4] identified home-based physical therapists as essential health care providers during the COVID-19 pandemic. Home-based physical therapy usually includes many approaches that make use of the outdoors around the patient’s home. However, the current situation is such that even outdoor approaches are difficult and activities may have to be carried out in confined spaces with ventilation. As the COVID-19 situation may be expected to continue, it is necessary to seek a safe and efficient approach that can prevent adverse effects on physical function and athletic performance as much as possible, even when activities are restricted.

Myofascial Release (MFR) is a manual approach to stretch the fascia [5,6]. So far, the main focus has been on improving joint mobility [6] and approaching pain [7], but in practice, in combination with exercise therapy, the indication has been expanded to include motor dysfunction [5], and now it is an approach aimed at improving pain and the effects of restricted exercise. Currently, MFR is mainly reported to have effects such as decreased pain, improvement in flexibility and range of motion, and improvement in depression and quality of life (QOL) [8]. In addition, this approach has the advantage that it can be carried out safely and efficiently [5,6,7,8,9]. However, although positive effects on dysfunction have been reported, there are no reports that MFR has improved physical performance. According to previous studies [10,11], physical performance is mainly gait speed, chair rise capacity, and balance skills which are evaluated on the lower limbs, and which are greatly affected by the strength of the quadriceps and hamstrings. Furthermore, the flexibility of the hamstrings is important because knee extensor torque is affected by the flexion angle of the hip joint [12]. As such, the flexibility of hamstrings is indispensable for improving physical performance.

In this study, it was decided to examine whether MFR for hamstrings, which is safe, efficient, and not burdensome to subjects, has a positive effect on physical performance. If it is possible to improve physical performance with MFR, home physiotherapy may effectively prevent deterioration of physical performance even in situations where physical activity is reduced or in a limited space.

## 2. Materials and Methods

### 2.1. Subjects

Seventeen healthy males and females volunteered to participate in this study: 10 males: age 20, height 170.2 ± 5.2 cm, weight 63.0 ± 9.9 kg; and 7 females: age 20, height 159.6 ± 3.7 cm, weight 52.6 ± 5.7 kg. Participants were not athletes, but general college students (not affiliated with university club sport activities or attending sports gyms). Participants who had any orthopedic issues (fractures, osteoarthritis, ligament injuries, pain, dysesthesia, etc.) or neurological abnormalities were excluded. Information about this study was provided in writing to all the participants prior to starting the assessment, and all participants provided their informed consent. This study was approved by the Yamato University Faculty of Health Sciences Research Ethics Committee (approval no. 48). MFR was conducted by one student who received one day of guidance from a physiotherapist who had worked clinically for more than 10 years. The MFR technique involves light contact over the skin, with the practitioner’s left palm on the participant’s medial hamstring and the right palm on the participant’s lateral hamstring. The practitioner continues to release the skin with light contact, to the extent that there is no slack in the skin. Care should be taken not to change the pressure of the hands (no pushing or stretching). MFR was performed according to the method of a previous study [13], once a day (3 sets for 60 s) for 5 consecutive days, on a bed in a prone position and relaxed state. Measurements were carried out 3 times, pre-intervention (Pre), immediately after MFR intervention (Immediately), and 5 days after continuous MFR intervention (Post 5 days).

### 2.2. Physical Measurements

We performed two physical function tests and five physical performance tests: finger floor distance (FFD), straight leg raising test (SLR), range of motion (ROM), standing long jump (SLJ), squat jump (SJ), functional reach test (FRT), and walking speed (maximum and comfortable speeds). A dedicated measurer was assigned to each measurement item.

The FFD test [14] used a measuring table with a height of 40 cm, and the participants stood on it for the measurement. The starting position was a static standing position on the table (step width 5 cm). After that, with participant’s own timing, participants were instructed to bend their trunk forward and to allow their upper limbs on both sides to hang down. The position of the midpoint on the line connecting the tips of the middle fingers on both sides was measured using a tape measure. The numerical values were measured in cm, with the height of the table set to 0 cm, and indicated by (−) when the fingers stopped at a position higher than the table surface and (+) when passing over the table surface. The measurement was performed only once, and the measurement was discontinued if the following events were found: (1) knee joint flexion was observed, (2) only one side upper limb was hanging down when the trunk was flexed, and (3) the trunk was extended or when participants bounced their stretches.

SLR measurements [14] were performed by two examiners. It was carried out using a goniometer according to the method of the Japanese Orthopedic Association and the Japanese Association of Rehabilitation Medicine [15]. Each participant was in a supine position on a bed, one of the two examiners raised the lower limb on the measurement side of the subject, and the other examiner adjusted the goniometer to the basic axis and the movement axis in 5° increments. The lower limbs were measured on both sides of the right SLR (SLR-r) and the left SLR (SLR-l), and each measurement was performed once.

SLJ was measured by two examiners. A tape measure was used for the measurement. Each participant’s start position was barefoot, the toes on both sides were aligned with the start line, and the step width was an arbitrary standing position. After that, they were instructed to jump forward as far as possible with arbitrary timing. There were no restrictions on the posture during movement or the swing of the upper limbs. Two examiners measured the straight-line distance of the heel (the heel near the start line) at the time of landing from the midpoint of the line connecting the toes on both sides of the start position. The unit was cm, and measurements were taken to the first decimal place. The measurement was performed twice, and that with the longer jumping distance was adopted. However, if (1) the foot moved at the time of landing, and/or (2) a body part other than the sole of the foot touched the floor at the time of landing, the measurement was discontinued. In addition, if both of the two measurements were discontinued, remeasurement was permitted once.

SJ was carried out based on the method of Coratella et al. [16]. The starting position was a posture in which the upper limbs on the wall side were extended upward so as to be perpendicular to the floor while standing in a barefoot standing position perpendicular to the wall. A choke was attached to the tip of the middle finger, and the wall was touched at the start position of marking. After that, each participant was then instructed to flex their knees about 90° to rest and jump upwards. In addition, they were instructed to touch and mark the wall at the highest point in the jump. The measurement was discontinued if the following events were found: (1) bounced stretches were performed before the jump, or (2) the wall was touched by anything other than the tip of the middle finger. A parallel line to the floor passing through each of the marked points was drawn, and the distance between each straight line was measured. The measurement was performed twice, and the average value was calculated.

FRT was carried out according to the method of Duncan et al. [17]. The starting posture of the participants was as follows: First, the shoulder joint on the measurement side was flexed 90° to the finger extension position, the elbow joint extension position, and the forearm intermediate position. After that, the standing posture was maintained so that the upper limbs were parallel to the wall. In addition, the position of the tip of the middle finger of the raised upper limb was marked on the wall. After that, participants were asked to reach as far forward as possible and mark the position of the tip of the middle finger again. A vertical line to the floor passing through each of the marked points at the start position and the marked points at the time of reach was drawn, and the distance between the straight lines was measured. The unit was cm, and the measurement was discontinued when a step was made when reaching or when a part of the body touched the wall.

For walking speed, each participant’s comfortable walking speed (C-walking speed) and maximum walking speed (M-walking speed) were measured. The measurement was performed by the same measurer using a stopwatch. The start and end of the measurement were taken when the participants’ torso reached the start and end points of the measurement section. In addition, the measurer performed the measurement in parallel with the walking of the participants. The measurement section was set to 20 m, and spare paths of 3 m were provided before and after the measurement section. Speed was measured twice, averaged, and then converted into a speed (m/s).

### 2.3. Statistical Analysis

All values are reported as means ± SD. Differences in continuous variables among the three time periods (Pre, Immediately, Post 5 days) were tested with repeated measures ANOVA of variance and corrected with Bonferroni’s post-hoc test. The significance level was set at *p* < 0.05. Statistical analysis was performed using IBM SPSS version 26.

## 3. Results

The characteristics of the participants are shown in Table 1. The results of the comparison of FFD, SLR-r, SLR-l, SLJ, SJ, FRT, comfortable walking speed, and maximum walking speed Pre, Immediately, and Post 5 days are shown in Table 2. Repeated measures ANOVA was performed on the 17 subjects who completed all measurements and three time periods. From repeated measurement ANOVA, FFD (Pre vs. Immediately vs. Post 5 days; −2.6 ± 8.9 vs. 0.4 ± 9.4 vs. 2.4 ± 8.9, *p* < 0.01), SLR-r (Pre vs. Immediately vs. Post 5 days; 76.2 ± 15.1 vs. 81.5 ± 12.7 vs. 80.3 ± 13.6, *p* = 0.02), SLJ (Pre vs. Immediately vs. Post 5 days; 185.6 ± 44.5 vs. 185.0 ± 41.8 vs. 196.6 ± 40.1, *p* < 0.01), and M-walking speed (Pre vs. Immediately vs. Post 5 days; 2.9 ± 0.6 vs. 3.0 ± 0.6 vs. 3.3 ± 0.6, *p* < 0.01) showed a significant change. There were no significant changes in the SLR-1, VJ, FRT, or C-walking speed variables. Bonferroni’s post-hoc test showed a significant increase between Pre vs. Immediately (−2.6 ± 8.9 vs. 0.4 ± 9.4, *p* < 0.05) and between Pre vs. Post 5 days (−2.6 ± 8.9 vs. 2.4 ± 8.9, *p* < 0.01) in FFD, between Pre vs. Post 5 days (185.6 ± 44.5 vs. 196.6 ± 40.1, *p* < 0.05) and between Immediately vs. Post 5 days (185.0 ± 41.8 vs. 196.6 ± 40.1, *p* < 0.01) in SLJ, and between Pre vs. Post 5 days (2.9 ± 0.6 vs. 3.3 ± 0.6, *p* < 0.01) and between Immediately vs. Post 5 days (3.0 ± 0.6 vs. 3.3 ± 0.6, *p* < 0.05) in M-walking speed.

## 4. Discussion

In this study, MFR was performed on hamstrings in 17 healthy men and women. The effect of MFR on physical performance was verified by measuring FFD, SLR, SLJ, SJ, FRT, C-walking speed, and M-walking speed at three periods of Pre, Immediately, and Post 5 days.

From Table 2, a significant increase in FFD was observed from Pre to Immediately, and from Pre to Post 5 days. Both FFD and SLR are tests that show the flexibility of hamstrings, but in this study, only FFD was significantly increased. In a previous study [18], it was confirmed that stretching the hamstrings improved FFD, and similar results were obtained in this study. Regarding SLR, no significant improvement was observed, but there was a tendency for the ROM to increase.

In SJ, there was no significant change in any of the periods compared to Pre. It is already known that SJ is mainly concerned with the strength of the muscles in the lower limbs [19,20]. From the result of this study, it seems that the MFR on the hamstrings could not improve the strength of the quadriceps, which is the knee extensor muscle, so that the vertical jump was not improved. Due to the duration of the study (5 days) and the fact that it was not aimed at participants with muscle weakness, it did not result in muscle strength gains. No significant changes were observed in SLJ and M-walking speed from Pre to Immediately, but improvement was observed after 5 days of continuous MFR. First, in terms of walking speed, Whitehead et al. [21] reported that simulated hamstring shortening produced adverse effects in the gait of normal subjects. Furthermore, as a result, it was reported that increased effort of walking (PCI) decreased speed, stride and step length. These results suggest that the flexibility and mobility of the hamstrings affect walking speed. However, in this study, there was no change in C-walking speed, and only an improvement in M-walking speed was observed. This may be related to the specificity of walking. Flexibility of the hamstrings is important because increased walking speed requires more mobility of the hip and knee joints [22]. In this study, FFD was significantly improved, but SLR was not significantly improved. SLR of this study was measured so that compensatory motion did not occur (compensatory motion: the contralateral thigh was lifted from the bed). Compensatory motion occurs due to the backward tilt of the pelvis due to the stretched hamstrings. Because of this precaution, it is considered that the hamstrings were measured in an unstretched state. SLR may have been a measurement of only mobility, not flexibility. In addition, Tully et al. [23] report that FFD reflects the extensibility of the hamstrings, with hip flexion more involved than spinal flexion as the fingers approach the toes. On the other hand, FFD is measured by automatic movement, and it seems that the weight of the trunk makes it easier for the pelvis to tilt forward, and the hamstrings are stretched. As such, it is considered that MFR improved the flexibility of the hamstrings as a result of FFD, and increased the motility of the hip and knee joints, which improved the M-walking speed as it requires the flexibility of the hamstrings. On the other hand, C-walking requires more efficient walking than speed, so it is possible that the flexibility of the hamstrings did not affect it. This suggests that M-walking requires deliberate effort, and therefore hamstring flexibility may have an effect on the increase in speed, but C-walking speed may not require as much hamstring flexibility.

In SLJ, it is possible that the increased flexibility of the hamstrings affected the SLJ’s start position and crouching motion [24], resulting in improved jumping distance. However, in this study, it cannot be determined whether the flexibility of the hamstrings affected the motion of SLJ, because motion analysis was not performed. From the SJ results, it is unlikely that the increase in SLJ and M-walking speed was due to the increase in quadriceps torque due to the increased flexibility of the hamstrings.

In this study, it was clarified that MFR for hamstrings affects not only the improvement in flexibility but also the improvement in physical performance of M-walking speed and SLJ. Currently, due to the influence of COVID-19, activity is restricted, so it may not be possible to actively go out and increase physical activity. However, it is important to perform MFR to maintain physical function and performance until the restriction of activity is lifted and people are able to actively increase the amount of physical activity. Furthermore, as muscle mass in the lower limbs decreases with aging [25], it is also necessary to prevent and maintain functional and motor decline in the lower limbs. MFR and physical activity have also been shown to have a positive effect on psychological factors such as depressive symptoms [8,26], so a synergistic effect may be expected. Furthermore, the assessment of walking speed in healthy young people is likely to benefit from the use of M-walking rather than C-walking.

The limitations of this study include the following: (1) the subjects were healthy young people, so it was not possible to determine if the effects would be similar in people with pain or in the elderly; (2) the effects on maximal muscle strength, gait, and SLJ movements could not be determined because muscle torque and movement analysis were not performed; (3) the study was conducted for a short period of time (5 days); and (4) the results could not be clarified because of the lack of a control group and the small sample size. In the future, it will be necessary to expand the sample size and participant conditions after setting a control group, and to analyze the muscle torque of the quadriceps femoris and changes in its movement over a long period of time by three-dimensional motion analysis.

## Figures and Tables

**Table 1:** Subjects’ characteristics.

Variable	All Subjects (n = 17)	Male (n = 10)	Female (n = 7)
Age, yr	20 ± 0.0	20 ± 0.0	20 ± 0.0
Height, cm	165.8 ± 7.2	170.2 ± 5.5	159.6 ± 4.0
Weight, kg	58.7 ± 10.2	63.0 ± 10.5	52.6 ± 6.2
BMI, kg/m^2^	21.3 ± 2.7	21.7 ± 3.0	20.7 ± 2.5

Values are presented as means ± SD. Abbreviations: BMI, body mass index.

**Table 2:** Effects of MFR intervention on physical function and performance (n = 17).

Variable	Pre	Immediately	Post 5 Days	*p*
FFD, cm	−2.6 ± 8.9	0.4 ± 9.4	2.4 ± 8.9	<0.01 *^,††^
SLR-r, °	76.2 ± 15.1	81.5 ± 12.7	80.3 ± 13.6	0.02
SLR-l, °	76.2 ± 15.0	81.8 ± 16.8	80.6 ± 15.6	n.s
SLJ, cm	185.6 ± 44.5	185.0 ± 41.8	196.6 ± 40.1	<0.01 ^†,‡‡^
SJ, cm	43.0 ± 11.3	43.9 ± 11.5	44.1 ± 10.6	n.s
FRT, cm	32.3 ± 6.0	33.4 ± 6.1	34.2 ± 5.6	n.s
C-walking speed, m/s	1.5 ± 0.2	1.5 ± 0.2	1.6 ± 0.2	n.s
M-walking speed, m/s	2.9 ± 0.6	3.0 ± 0.6	3.3 ± 0.6	< 0.01 ^††,‡^

Values are presented as means ± SD. Abbreviations: Pre, pre-intervention; Immediately, immediately after MFR intervention; Post 5 days, 5 days after continuous MFR intervention; MFR, Myofascial Release; FFD, finger floor distance; SLR-r, straight leg raising—right; SLR-l, straight leg raising—left; SLJ, standing long jump; SJ, squat jump; FRT, functional reach test; C-walking speed, comfortable walking speeds; M-walking speed, maximum walking speeds; n.s., not significant. Repeated measurement ANOVA (with post-hoc Bonferroni): * Pre vs. Immediately, <0.05; ^†^ Pre vs. Post 5 days, <0.05; ^††^ Pre vs. Post 5 days, <0.01; ^‡^ Immediately vs. Post 5 days, <0.05; ^‡‡^ Immediately vs. Post 5 days, <0.01.

## Data Availability

Not applicable.
